# Guided Embodiment and Potential Applications of Tutor Systems in Language Instruction and Rehabilitation

**DOI:** 10.3389/fpsyg.2018.00927

**Published:** 2018-06-13

**Authors:** Manuela Macedonia, Florian Hammer, Otto Weichselbaum

**Affiliations:** ^1^Information Engineering, Johannes Kepler Universität Linz, Linz, Austria; ^2^Neural Mechanisms of Human Communication, Max-Planck-Institut für Kognitions- und Neurowissenschaften, Leipzig, Germany; ^3^Linz Center of Mechatronics GmbH, Linz, Austria; ^4^Sew Systems Gmbh, Linz, Austria

**Keywords:** tutor systems, language instruction, aphasia therapy, intelligent tutor system, gesture production, gesture recognition, learning

## Abstract

Intelligent tutor systems (ITSs) in mobile devices take us through learning tasks and make learning ubiquitous, autonomous, and at low cost (Nye, 2015). In this paper, we describe guided embodiment as an ITS essential feature for second language learning (L2) and aphasia rehabilitation (ARe) that enhances efficiency in the learning process. In embodiment, cognitive processes, here specifically language (re)learning are grounded in actions and gestures (Pecher and Zwaan, 2005; Fischer and Zwaan, 2008; Dijkstra and Post, 2015). In order to guide users through embodiment, ITSs must track action and gesture, and give corrective feed-back to achieve the users' goals. Therefore, sensor systems are essential to guided embodiment. In the next sections, we describe sensor systems that can be implemented in ITS for guided embodiment.

Today in L2 learning, ITSs transpose classroom activities as reading, listening, and making exercises in electronic environments (Holland et al., 2013). Similarly in ARe, a virtual therapist in a tablet helps patients in the treatment of verbal anomia by presenting pictures (Lavoie et al., 2016). Virtual therapists do basically what a human therapist would do, i.e., they ask patients to name the pictures presented (Brandenburg et al., 2013; Kurland et al., 2014; Szabo and Dittelman, 2014).

Both domains, L2 and ARe, still treat language a purely mentalistic process, a manipulation of symbols in our minds (Fodor, 1976, 1983). Consequently, symbols such as written words or pictures representing the word's semantics are the base of main stream language educational and rehabilitation methods. Despite this, in the last three decades, a growing number of studies have converged to suggest that language as a cognitive capacity is grounded in our bodily experiences in the environment, in perception and action (Lakoff, 2012; Dijkstra and Post, 2015; Borghi and Zarcone, 2016). Words are not symbols any more. Instead, they have been described as “experience related brain networks” (Pulvermüller, 2002). Interestingly, not only concrete but also abstract vocabulary is rooted in the body. In a comprehensive review of neuroscientific studies, Meteyard and colleagues show that simple recognition of abstract words elicits activity in sensorimotor brain regions (Meteyard et al., 2012). This is explained by the fact that abstract concepts are also internalized by real experiences that in their turn are related to the body. Take for example the word *love*: it is embodied because acquired from concrete and experienced concepts, i.e., perceiving the partner physically, doing things with the partner, and so on. All these experiences converge to a metaphorical extension which is labeled as *love*.

In fact, first language acquisition is tightly connected to sensorimotor experiences (Inkster et al., 2016; Thill and Twomey, 2016). In infancy, the body is the main vehicle that collects experiences related to language units as nouns and verbs (Tomasello et al., 2017). Furthermore, very early in development, gestures make their appearance. They are precursors of spoken language (Mattos and Hinzen, 2015) and tightly bound to it. Language and gestures represent the two sides of the human communicative system (Kelly et al., 2010).

In adult age, the body can be used as a tool to enhance memory for verbal information (Zimmer, 2001). This is achieved by performing gestures to words or phrases that are to be memorized. The effect of gestures on memory for verbal information has been named “enactment effect” (EE) Engelkamp and Zimmer (1985) and “self-performed task effect” (Cohen, 1981). The EE is robust and has been extensively investigated with different materials, tests, and populations (Von Essen and Nilsson, 2003). In memory research, the EE effect has been reconducted to a motor trace that the gesture leaves in words' representations (Engelkamp, 1998).

Also, in second language learning, self-performed gestures accompanying words enhance memory performance compared to just reading the words and/or listening to them (Macedonia, 2014), in the short and in the long term (Macedonia and Klimesch, 2014). In a study with functional Magnet Resonance Imaging (fMRI), Macedonia and Mueller (2016) have shown that passive recognition of second language words trained with gestures activates extended sensorimotor networks. These networks involve motor cortices and subcortical structures as the basal ganglia, and the cerebellum. They all participate to a large motor network. It is thus conceivable that retention is superior because words learned with gestures might engage procedural memory in addition to declarative memory (Nilsson and Bäckman, 1989). Interestingly, recent studies on patients with impaired procedural memory have demonstrated that the patients could not take advantage of learning through gestures (Klooster et al., 2014).

In aphasia, gestures produced by patients trying to communicate can easily be observed. These gestures fulfill compensatory functions (Göksun et al., 2015; Rose et al., 2016) if the patients' language is impoverished or omitted (Pritchard et al., 2015). However, because of the high variance in lesion patterns, age of the patients, patho-linguistic profile, intensity of intervention, etc., studies employing gestures and studies employing other therapeutic instruments are difficult to compare. Hence, effects of gestures on rehabilitation can be diverging (Kroenke et al., 2013). Main stream aphasia therapy is still constrained to the verbal modality and bans gestures as tool that might help to restore language networks (Pulvermüller, 2002). Nevertheless, a growing number of studies show that action and gesture can help support the missing side of the communicative coin (Rose, 2013). Whereas simple observation of action has a positive impact on word recovery (Bonifazi et al., 2013), observation followed by execution of action leads to better recovery results (Marangolo and Caltagirone, 2014). These studies pave the way for a novel understanding of aphasia therapy in which the body helps the mind to regain language functions, as long as brain structures serving procedural memory are not compromised (Klooster et al., 2014).

This is to say that humans need the body to acquire first language, to support memory for verbal information, to learn a second language, and to reacquire language functions disrupted by brain lesions. At this point, a core issue is to stress that embodiment of language needs active experience. In enactment research, it has long been known that it is not enough to observe gestures and actions, one must perform them (Cohen, 1981; Engelkamp et al., 1994). When interacting with an ITS, the user is first presented with the language to be trained and the gestures to be performed. Thereafter, the user must perform the actions and the gestures. Monitoring can make action performance accurate in execution. Thus, one component of the ITS must detect motion and gesture, compare it with a template and give feed-back on execution accuracy. Execution monitoring needs sensor systems.

## Technologies for gesture performance monitoring

**Figure 1:**
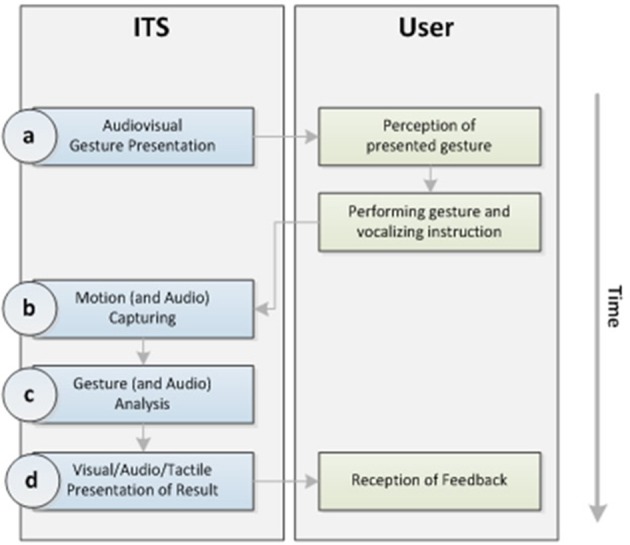
Embodiment interaction model.

Guided embodiment requires an interaction between ITS and user: A gesture representing a concept is performed by an ITS avatar. The user observes the gesture and imitates it. The user's gesture must be sensed during performance. Performance is evaluated by the system on the base of a template. Visual, auditory and or tactile feedback is given by the ITS (please see Figure 1).

### Audio-visual gesture presentation (AVGP)

First, a written word is presented to the user on a display simultaneously with a video in which an actor performs a representational gesture. The gesture can be presented by a human through a video or by an avatar, or an agent (Bergmann, 2015). Synchronously, an audio file of the word is played via loudspeaker.

#### Motion capturing

Motion is the change of body position in time. Motion capturing occurs as a two-phases process. First, a single motion is sensed generating data (*motion sensing*) (Moeslund et al., 2006). Secondly, the data are sampled (*motion sampling*) and sequenced in time into a movement path, a so-called motion trajectory model. Depending on the location of the sensors used to detect the motion, Motion capturing can be subdivided into two categories: *infrastructure based* or through *wearables*. Infrastructure-based systems rely on hardware that is rigidly mounted inside a room as high-speed infrared cameras in a gait analysis laboratory, or sensors in a blue screen environment. Infrastructure based systems use sensors with high power consumption.

Systems based on **microwave, ultrasonic or radar sensors** operate by emitting electromagnetic or sonic waves and sensing the echo received. Depending on the purpose of motion capturing, sensor technologies can vary. For example, ultrasonic motion detection is quite common in prenatal diagnostics (Birnholz et al., 1978). For remote vital sign detection radar-based motion detection is frequently used (Lubecke et al., 2002).

**Vision-based systems (VBS)**, including single camera, multiple cameras, and depth camera systems, play the most important role in human motion capture. Sensors detect light which can be visible or invisible to the human eye which is emitted or reflected by the body or an object (Moeslund et al., 2006).

**Single camera-based motion detection systems** are present in notebooks, tablets, and mobiles. Although these systems often have a high-quality resolution, they operate with a single camera. A single camera cannot capture the motion of body parts that are occluded by other body parts. This results in an inaccurate or incomplete analysis of the motion.

**Multiple camera systems** with two or more cameras allow 3D capturing. Algorithms combining 2D images from the cameras calculate a 3D-resolution (Aggarwal and Cai, 1997; Cai and Aggarwal, 1999). In the 3D-resolution, the synchronized recordings are combined. The combination includes the positions of the cameras relative to each other and their angles of view. Multiple camera systems are used in rigid mounted setups, in laboratories or dedicated rooms for example in rehabilitation (gait analysis), and sports (motion analysis).

**Depth cameras** sense 3D-information by means of infrared light. They calculate the distance between the camera and a body in two ways. They project an invisible grid onto the scenery and sense the grid's deformations. Alternatively, they measure the distance to the scenery and they calculate the transfer time of the infrared light from the camera to the object. This second kind of depth camera is also called “Time-of-flight”-camera (ToF) (Barnachon et al., 2014; Cunha et al., 2016; Garn et al., 2016).

Depth camera systems with a single device do not overcome the problem of occluded parts (Han et al., 2013). However, they have an advantage: they provide information about the distance of each object or body within the camera's view relative to the camera's position. These systems do not rely on heuristics about proportions of the object in order to determine its distance. This information increases accuracy in calculating the position of a human body or object.

**Wearables** are sensors worn on the body. They are light-weighted and have low power consumption. They are often used in sports (Roetenberg et al., 2013). Among wearables, we find inertial measurement units (IMUs) and sensing textiles.

**Inertial Measurement Units (IMUs)** are small electronic devices that measure acceleration, angular changes and changes in the magnetic field surrounding the body or object (Roetenberg, 2006; Shkel, 2011). If the starting position is known, an approximate position at time *t* is can be calculated by implementing the changes in forces, angles and magnetic field from the starting position up to *t*. IMUs differ from camera-based systems: while the latter measure the absolute position of the body at every time point *t*, IMUs acquire a starting position and the movement's sequence.

IMUs are integrated into wearable objects and respond on minimal deviations of the sensors by showing a drift. This drift can sum up to false positions over time. Fusion algorithms combining filtering and validation of sensor are used to compensate, respectively minimize drifts values (Luinge and Veltink, 2005; Sabatini, 2011; Roetenberg et al., 2013).

**Sensing textiles** represent a novel way of capturing motion. They consist of fabrics containing enwoven pressure sensitive fibers. These fibers change their electric resistance depending on the pressure changes that they sense (Mazzoldi et al., 2002; Parzer et al., 2016). Clothes tailored with these fabrics enable to calculate movements of the body in a fine-grained way (Parzer et al., 2016). The choice of the adequate type of motion sensing technology depends on the application domain. In our case, sensing of human body movements for an ITS can be accomplished with four sensor technologies: camera, depth-camera, IMUs, and sensing textiles.

Vision-based systems (VBS) take pictures over time and analyze them in order to detect body parts. Thereafter, VBS transform the detected body parts into digital representations, into human body models. Common models are skeletal, joint-based (Badler and Smoliar, 1979; Han et al., 2017), and mesh-based (de Aguiar et al., 2007). For an overview and classification of the major techniques used for sampling 3D data, please see Aggarwal and Xia (2014).

Additionally, VBS can increase the accuracy of the human body model by markers as light-emitting diodes, passive reflectors or patterns. These markers are fixed on pre-defined body parts and map them to the according representation within the model. Marker-less systems use heuristics about shapes, dimensions, and relations between body parts estimating and calculating the model according to these constraints.

Body data are sampled and thereafter transferred into a digital form in constant periods of time. This is done in order to obtain the motion trajectory model needed. It represents the body parts and their changes in posture over the time of recording (Poppe, 2010). Hence, motion sampling results in a motion trajectory model.

### Gesture (and audio) analysis

In the literature, different approaches for matching motion trajectory models are discussed. Kollorz et al. (2008) ground their model on projections of image depth. Mitra and Acharya (2007) describe the use of hidden Markov models (Rabiner and Juang, 1986), finite-state machines (Marvin, 1967) and, neural networks (Lippmann, 1987). Other authors use a support-vector machine-based approach (Cristianini and Shawe-Taylor, 2000; Schuldt et al., 2004; Miranda et al., 2014). A template-based method for matching motion has been developed by Müller and Röder (2006). Stiefmeier et al. (2007) convert the motion trajectory model into strings of symbols. This is done in order to apply string matching algorithms that are faster in running analyses. Detailed reviews on vision-based human motion recognition methods are provided by Poppe (2010) and Weinland et al. (2011).

Embodiment-based ITS employed in language learning and rehabilitation need real-time processing of sensed gestures because of the immediate feedback on gesture accuracy that users need (Ganapathi et al., 2010).

Accuracy in sound reproduction is an important issue in both, second language learning and aphasia rehabilitation. Language output by the user is recorded and analyzed by different methods (Rabiner and Juang, 1993). Recent approaches employ complex models as neural networks for speech recognition (Hinton et al., 2012; Graves et al., 2013).

After a match between the sensed gesture or the voice and the template within the representing motion trajectory model has occurred, feedback can follow. It can be visual via the display, acoustical with sound through a speaker (built-in or external), and tactorial by means of a vibration given by the device. Feedback can be simple (i.e., a sound or synthesized speech).

### Evaluation of sensor technologies

In order to give an overview of the sensor technologies presented in the preceding sections, we created Table 1. It describes the degree of following characteristics: accuracy in motion sensing, ease of set up for an expert, mobility and size. Note that the description is done for the use of a professional (lab technician) and for an institution (language school or hospital). We do not consider ITS software, software processes, and design patterns, or aspects of user-interface design. For further reading, please see (Oppermann, 2002; Dillon, 2003; Carroll, 2006; Smith-Atakan, 2006; Preece et al., 2015).

**Table 1:** Evaluation of sensor technologies.

	**Singlecamera**	**Multiplecameras**	**Depthcamera**	**Sensingtextiles**	**IMUs**
Accuracy	0	++	+	++	0
Setup	++	+	+	++	+
Mobility	+	+	+	++	++
Size	+	+	0	++	+

*0, moderately fulfilling the users' requirements; +, fulfilling the requirements; ++, fulfilling the requirements very well*.

In this paper, we describe two application domains for ITS following principles of guided embodiment: language (re-)learning and aphasia rehabilitation. So far, we have focused on the possible use of the ITS in an institution (school vs. hospital). However, considering that language learning and rehabilitation need massed practice (Pulvermüller et al., 2001; Kurland et al., 2014), ITS should accompany users during the learning task in their homes. Sensing textiles can represent an emerging field in guided embodiment for language learning and aphasia rehabilitation. A learning t-shirt could combine a few advantages: high accuracy in sensing motion, ease of use and possible vibration feedback. However, to our knowledge no such system is present to date on the market, even as a prototype.

To present, only single camera systems present in tablets and mobile phones are affordable and easy to use. Also, nearly everyone has an own device. Because of their size, single camera systems can be carried where users need them. Despite the fact that presently single cameras are not very accurate in motion capturing as described in the preceding section, they might become the instruments used in a near future.

Altogether, this brief overview highlights the fact that guided embodiment of language could be the way to enhance performance in learning and rehabilitation. However, more research in the field is needed.

## Author contributions

MM has laid down the structure of this paper and written the sections on embodiment. FH and OW have written the sections on technologies for gesture performance monitoring.

### Conflict of interest statement

The authors declare that the research was conducted in the absence of any commercial or financial relationships that could be construed as a potential conflict of interest.
